# Unravelling potential reaction intermediates during catalytic pyrolysis of polypropylene with microscopy and spectroscopy†

**DOI:** 10.1039/d3cy01473h

**Published:** 2024-01-11

**Authors:** Ina Vollmer, Michael J. F. Jenks, Sebastian Rejman, Florian Meirer, Andrei Gurinov, Marc Baldus, Bert M. Weckhuysen

**Affiliations:** a Inorganic Chemistry and Catalysis group, Debye Institute for Nanomaterials Science and Institute for Sustainable and Circular Chemistry, Department of Chemistry, Utrecht University Universiteitsweg 99 3584 CH Utrecht The Netherlands b.m.weckhuysen@uu.nl; b NMR Spectroscopy, Bijvoet Center for Biomolecular Research, Department of Chemistry, Utrecht University Padualaan 8 3584 CH Utrecht The Netherlands

## Abstract

While plastics-to-plastics recycling *via* melting and re-extrusion is often the preferred option due to a relatively low CO_2_ footprint, this technique requires a highly sorted waste stream and plastic properties can often not be maintained. Obtaining aromatics, such as benzene, toluene, and xylene (BTX), *via* catalytic pyrolysis of polyolefins, such as polypropylene and polyethylene, offers another attractive recycling technology. In this process, a discarded crude oil refinery catalyst (ECAT) was previously shown to lower the unwanted formation of deactivating coke species compared to a fresh crude oil refinery catalyst (FCC-cat), while yielding 20 wt% aromatics from polypropylene. In this work, we study the underlying reaction mechanism for this chemical recycling process over the fresh and used refinery catalyst as well as a model system, not containing any zeolite material, using a combination of microscopy and spectroscopy. More specifically, by using *in situ* fluorescence microscopy, *in situ* infrared spectroscopy, *in situ* ultraviolet-visible spectroscopy as well as *ex situ* solid-state nuclear magnetic resonance, we observe highly fluorescent methylated aromatic intermediates that differ for the three catalyst materials under study both in their fluorescence, IR, UV-vis, and NMR spectroscopy features. This detailed micro-spectroscopic comparison informs which potential reaction intermediates lead to increased coke formation. Our results suggests that a next generation of catalyst materials for this process would profit from a higher accessibility and a milder acidity compared to an FCC-cat and shows the great potential of using ECAT to reduce coking and obtain a BTX stream, which could be become the chemical building blocks for the manufacturing of *e.g.*, plastics and coating materials.

## Abstract 2

Confocal fluorescence microscopy, infrared spectroscopy, UV-Vis spectroscopy and nuclear magnetic resonance are used to study the reaction intermediates during cracking of polypropylene over a fresh and used fluid catalytic cracking catalyst.

## Graphical abstract

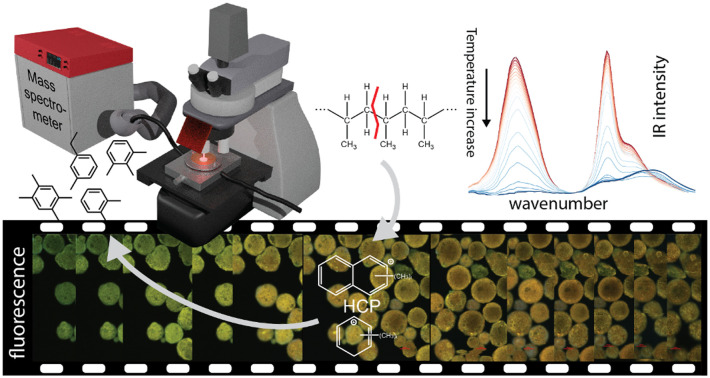

## Introduction

As an addition to the more traditional plastic recycling *via* melting and re-extrusion or mechanical recycling, chemical recycling of plastics could help boost global recycling rates from currently only 12 wt%.^1^ Some sources predict chemical recycling can serve as a facilitator of major profit-pool growth for the petrochemical industry, contributing 17% to the overall recycling volume,^2^ others see its potential as fill-in specifically for food-grade applications.^3^ While its exact role is still up for discussion, the need for additional and alternative recycling technologies is undisputed.^1^ Reversing the polymer making process, converting plastics back to its constituting monomers, is appealing as it allows for plastics-to-plastics recycling and thus in principle also a circular economy for plastics. While this is achievable with solvolysis techniques, these are only applicable to polyesters, polyamides, polyurethanes and polycarbonates, which contain heteroatoms in the backbone and thus provide a clear chemical bond for the cleavage agent to act selectively at comparatively mild temperatures. Other polymers require higher temperatures and of these only polymethylmethacrylate (PMMA) and polystyrene (PS) have large enough pendant groups to allow for selective cleavage to monomers with reasonable yields. However, monomer recovery from polypropylene (PP) and polyethylene (PE) conversion is very difficult.^4^ Aromatics, *i.e.*, benzene/toluene/xylene (BTX) mixtures, have a comparative market value^5^ and market volume as the monomers ethylene and propylene and have the additional benefit of being transportable without compression.^6^ BTX are also used to make monomers for PS and polyethylene terephthalate (PET) manufacturing. However, a significant yield of aromatics cannot be obtained by mere thermal conversion of polyolefins.

In our previous work, we have employed a fluid catalytic cracking (FCC) catalyst material, further denoted as FCC-cat, currently used for the conversion of vacuum gas oil (VGO) into gasoline and light olefines, such as propylene. The main cracking and aromatization activity of the FCC-cat composite material is associated with the zeolite Y component, while silica and alumina in the catalyst matrix pre-crack larger feed molecules and clay is mainly used to shape the catalyst particle into 50–150 μm spheres. During operation, the FCC-cat undergoes repeated cycles of reaction, deactivation and regeneration, establishing a catalyst particle age distribution in the reactor/regenerator system. The bulk of this catalyst forms the equilibrium catalyst (ECAT) and fractions of it are removed at regular time intervals, while new FCC-cat is regularly added to maintain the required process efficiency and a certain product distribution. This is necessary, because of irreversible catalyst deactivation due to heavy steaming of the zeolite component of the catalyst material in the regenerator. In addition, metals, such as Fe, V, and Ni, originating from the crude-oil based VGO and reactor fouling, deposit on the catalyst during the process.^7–14^ Other authors have already demonstrated the potential of the FCC-cat for polyolefin conversion obtaining mainly C1–C4 gaseous hydrocarbons and gasoline range alkanes and alkenes.^15–17^

In our previous work, PP was fully converted into a gasoline-like product containing ∼20 wt% of aromatics in a batch reactor with a catalyst-to-polymer ratio of 1 : 2.^18^ Contrary to our work, only 2 wt% combined BTX yield was achieved previously, likely due to a different reactor geometry, *i.e.* a fluidized bed, and thus a shorter contact time of the polymer with the catalyst.^15^ Interestingly, we found that ECAT discarded from the FCC process produced the same share of aromatics, while we found less deactivating coke species on the ECAT after reaction compared to the fresh FCC-cat. Other authors have also found a higher stability of the ECAT upon several regeneration cycles.^17^ In addition, the matrix of the FCC-cat itself can lead to significant formation of aromatics as we showed previously using a FCC-cat without the zeolite additive.^18^

It is estimated that on average 0.16 kg of ECAT is discarded per barrel of crude oil converted,^19^ with an installed FCC capacity of over 14 million barrels of crude oil per day.^20^ While the discarded catalyst shows some potential to be recycled as cement additive,^21^ it can be considered a waste product and its use for plastic waste conversion could improve the overall economics of such a catalytic process of plastic recycling. Ideally, however, such a catalytic pyrolysis process would produce a pure stream of mono-aromatics from plastic waste.

To achieve this goal, more fundamental knowledge about the underlying mechanism of polyolefin conversion over solid acid catalysts is necessary. So far, this mechanism is speculated to proceed *via* carbenium ions.^22^ However, not much is known about the steps following the activation of the polymer and additional possible intermediates of the overall chemical reaction process. For several other reactions that employ a zeolite catalyst, most prominently the methanol-to-hydrocarbon (MTH) reaction, a hydrocarbon pool (HCP) made up by charged aromatic species was found to play an important role and was influenced by metal addition to the zeolite.^23–26^ To investigate the chemical nature of these HCP species, magic angle spinning (MAS) solid-state NMR (ss NMR) has been shown to be very powerful, revealing aliphatic and aromatic species. Previously, 2-D double quantum ^1^H ssNMR measurements have been used to reveal the correlation between aliphatic and aromatic protons, indicating the presence of methylated benzenes and naphtalenes.^25^

In this work, we investigated the reaction intermediates formed during catalytic pyrolysis of PP over FCC-cat, ECAT and a model catalyst equivalent to FCC-cat but without containing the zeolite, which is denoted as FCC-NZ. We use *in situ* infrared (IR) spectroscopy, *in situ* fluorescence microscopy, and *in situ* ultraviolet-visible (UV-vis) spectroscopy. In addition, we employ *ex situ* two-dimensional (2-D) (^1^H–^13^C) heteronuclear correlation (HETCOR) ssNMR spectroscopy at very high magnetic field (*i.e.*, 1.2 GHz).

## Results and discussion

The catalyst materials employed in this work are the same as in our previous publication and detailed characterization of their acidity and porosity can be found there.^18^ In addition, catalytic results obtained in a semi-batch reactor and a detailed product analysis can be found in that work. Here, we focus on the fundamental understanding of the underlying mechanism that leads to the observed product formation and coking behaviour. All experiments were performed in a Linkam reactor, equipped with CaF_2_ windows allowing for *in situ* microscopic and spectroscopic observations (Fig. S1†). The catalyst particles and small pieces of PP were placed on a heating stage and a 15 ml min^−1^ N_2_ flow sent over the catalyst/plastic mixture. The temperature was increased at 10 °C min^−1^. Ramped experiments have several advantages over isothermal experiments. It is easy to miss the onset of reaction under isothermal conditions as the polymer is already present in the cell during heating. It is conceivable that a dosing system for the polymer could be developed in the future to enable this kind of measurements, although rather complicated on such a small scale. To confirm the validity of our ramped experiments, we repeated a few experiments under isothermal conditions at 300 °C (Fig. S2 and S3†), which resulted in similar spectral features as discussed below for the ramped experiments.

**Fig. 1:**
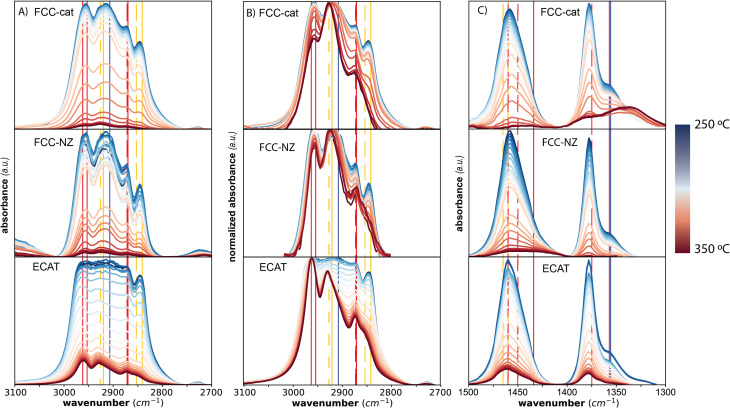
*In situ* infrared (IR) spectra measured during reaction of polypropylene (PP) with a fluid catalytic cracking catalyst (FCC-cat), a FCC-cat without zeolite (FCC-NZ) and an equilibrium catalyst (ECAT), while ramping the temperature at a rate of 10 °C min^−1^. Panel A shows spectra of the C–H stretching, while Panel B shows the normalized C–H stretching region to better visualize peak shifts. Panel C shows the C–H bending region. The lines indicate the vibrational frequencies of the chemical bonds highlighted in the PP structure shown in Scheme 1 (red – CH_3_, yellow – CH_2_ and blue – tertiary hydrogen), where dashed lines show the stretching representative for smaller hydrocarbons. Full non-normalized IR spectra without baseline subtraction can be found in Fig. S4.†

**Scheme 1:**
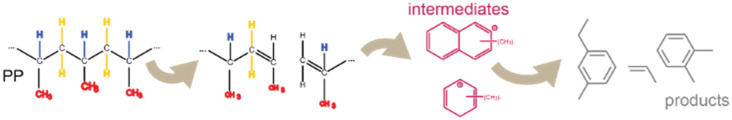
Polypropylene (PP) is cleaved to smaller hydrocarbons, which react further to (aromatic) intermediate from which final products are formed. Highlights of specific bonds correspond to the assignment in Fig. 1.

For *in situ* IR spectroscopy, we have focused on an individual catalyst particle. When the PP melts at ∼170 °C, the catalyst particle is engulfed in molten PP, which gives rise to a strong IR signal in the C–H stretching region (Fig. 1A and B), which saturates the detector initially, and a signal in the C–H bending region (Fig. 1C). The decrease in intensity in these regions upon heating the reaction mixture indicates the cleavage of the backbone C–C bonds of PP and the formation of smaller hydrocarbons, which leave as gas-phase products or are further converted to cyclic and aromatic products (Scheme 1). The smaller hydrocarbons give rise to slightly shifted peaks in the C–H stretching region. The symmetric C–H stretching of the methylene group (–CH_2_–) shifts from 2841 to 2853 cm^−1^ and the asymmetric C–H stretching from 2920 to 2926 cm^−1^.^27–29^ This is indicated by yellow lines in Fig. 1A and B, where the dashed lines correspond to the peak locations, which are typical for smaller hydrocarbons.

The ratio of intensities of peaks associated with the methylene and the methyl group (–CH_3_, red in Fig. 1A and B) decreases due to the formation of methyl chain ends after cleavage and the peak associated with tertiary hydrogen (blue lines in Fig. 1A and B) decreases in intensity. Notably, no peaks associated with aromatics were observed in the spectrum, likely due to a lack of sensitivity or overlap of absorption with the catalyst material.

**Fig. 2:**
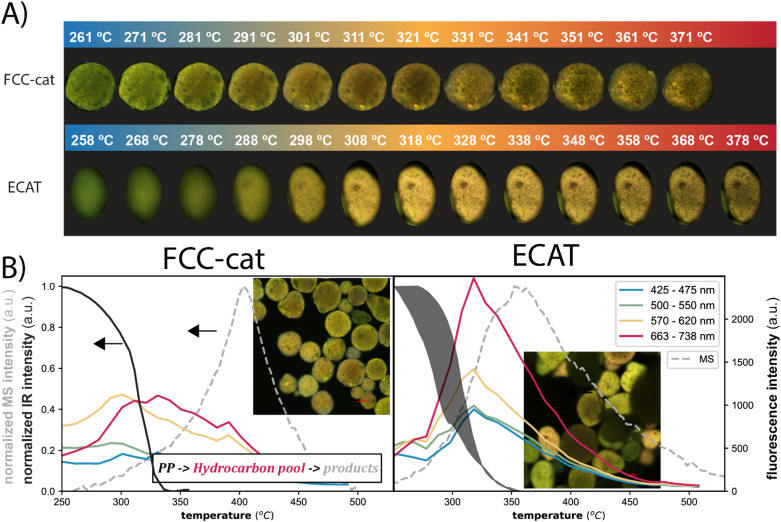
Panel A depicts the *in situ* fluorescence microscopy images of selected FCC-cat (top), ECAT (middle) and FCC-NZ (bottom) particles during the polypropylene (PP) catalytic pyrolysis reaction. All fluorescence microscopy images of all catalyst particles imaged can be found in Fig. S5.† Panel B: The integrated peak area of the C–H bending vibrations measured by *in situ* IR spectroscopy (Fig. 1C) indicates PP breakdown over FCC-cat (left), FCC-NZ (middle) and ECAT (right). The fluorescence intensity in the different wavelength regions is obtained by averaging over all pixels of a sectioned catalyst particle. Evolution of fluorescence for more ECAT particles can be found in Fig. S6.†

Confocal fluorescence microscopy (CFM) was previously used to study the accessibility and strength of acid sites on FCC-cat,^9,30,31^ ECAT,^32^ clay-bound ZSM-5-based catalyst bodies^33^ and various pure zeolites^34–38^ using fluorescent probes and studying chemical reactions *in situ* or *operando*. In this work, it was used to track aromatic species during the reaction. Several catalyst particles were imaged (Fig. S5†) with one minute between each scan. Corresponding temperatures are indicated above the images in Fig. 2A, which shows the evolution of fluorescence of single FCC-cat particles manually sectioned from the images of the CFM scans. Fluorescence was excited with four different lasers and detected in the corresponding wavelength regions creating an image of four channels. Only aromatic species are excited,^39^ while PP, alkanes and alkenes do not absorb light in the measured wavenumber regions.^40^ The fluorescence intensity first increases dramatically and then drops again with reaction progression. To visualize this more clearly, the average intensity in the different wavelength regions is plotted over the course of the reaction. The fluorescence is obtained by averaging over all pixels of the sectioned catalyst particles shown in Fig. 2A. The development of the average fluorescence is compared to the development in IR intensity in the C–H bending region, representative of C–C cleavage (black line in Fig. 2B). The IR intensity decreases first slowly and then faster. The steepest decline of the IR intensity signifies the point at which C–C cleavage rate is at its maximum. This occurs at around 325 °C.

**Fig. 3:**
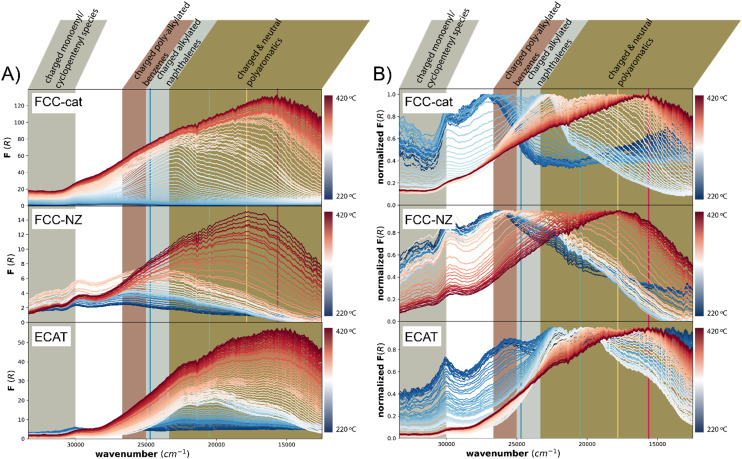
Panel A shows the spectra of the related *in situ* UV-vis spectroscopy measurements, while Panel B shows the normalized UV-vis spectra for FCC-cat (top), FCC-NZ (middle) and ECAT (bottom) particles during the polypropylene (PP) catalytic pyrolysis reaction, including the different spectroscopic assignments of the different absorption bands observed. The colored lines correspond to the wavenumbers of the lasers used for *in situ* confocal fluorescence microscopy (CFM).

As the fluorescent species arise as soon as the C–C cleavage is observed with IR spectroscopy they are aromatic intermediates and products, which form after the initial cleavage of the polymer (Scheme 1). The fluorescence intensity is the lowest for FCC-NZ and the highest for ECAT, which connects well with their respective activities (Fig. S7†). In addition, the intensity of the fluorescence arising from the red laser is the lowest for FCC-NZ, while it is the highest for FCC-cat. Fluorescence in this high wavelength region relates to the amount of polyaromatic species present and thus indicates less of those species for FCC-NZ, which is well in line with the significantly lower coking and aromatics production observed on FCC-NZ.^41^ The fluorescence for this catalyst is instead dominated by species excited by the green and the blue laser, which corresponds to smaller aromatic species. Over the FCC-cat, fluorescence is observed in two phases. Fluorescence peaks first for the yellow and green laser before the C–C cleavage rate is at its maximum, while the fluorescence from the species observed with the red laser peaks only when no more C–C cleavage is observed. This indicates a buildup of potential reaction intermediates on the catalyst, which are then further converted to either coke deposits or other products. Over the ECAT material, a much higher fluorescence was observed, which could indicate differences in the intermediates observed. In addition, the fluorescence intensity peaks earlier than for FCC-cat and also at the same temperature for all four lasers. This might be due to its higher activity and accessibility causing all reactions to occur simultaneously (Fig. S7†).^42^ To characterize the intermediates further, we have performed *in situ* UV-vis spectroscopy in the same reactor as for the experiments described above (Fig. 3).

This technique is complementary to fluorescence measurements as it represents the excitation part of the CFM measurement, while also probing the higher wavenumber range. For all catalysts under study, the overall intensity of the spectra increases over the course of the reaction (Fig. 3A), but the final absorption intensity of FCC-NZ and ECAT are only 12% and 52% of the intensity of FCC-cat. This is only in partial agreement with the CFM results, where FCC-NZ showed the lowest fluorescence intensity, in agreement with UV-vis absorption intensity, while ECAT shows a disproportionally high fluorescence. This could be explained by fluorescence quenching on FCC-cat due to the formation of graphitic structures.^43^ The order in which fluorescence appears during the conversion phase detected with IR spectroscopy is in line with the time evolution of the UV-vis absorption spectra, however once all polymer was converted, at ∼250 °C, the absorption intensity still increases in the UV-vis spectra, while the fluorescence intensity decreases. This could again be due to fluorescence quenching when more and more graphitic coke species are formed. The UV-vis absorption feature observed at ∼30 000 cm^−1^ for all three catalysts arises from the catalyst material itself (Fig. S8†). Interestingly, opposite to what is observed for the MTH process, almost no charged monoenyl/cyclopentenyl species are observed. The feature at ∼27 000 cm^−1^, attributed to charged poly-alkylated benzenes, appears at 220 °C before any conversion is observed with IR and then gradually shifts to lower wavenumbers representative of more conjugated species that likely act as precursor to coke. This transformation is much slower for FCC-NZ than for FCC-cat, while initial C–C cleavage was observed at similar temperatures for both catalysts with IR. The spectral shape of the three catalysts shows remarkable differences. A distinct feature at ∼22 000 cm^−1^, in the region corresponding to charged and neutral polyaromatics, appears at 280 °C for FCC-cat, while on FCC-NZ the feature is much broader and its maximum reaches the same wavenumber only at 355 °C. This could be attributed to the missing zeolite domains on FCC-NZ.

On ECAT two additional features appear at ∼19 500 cm^−1^ and ∼16 625 cm^−1^ and these features appear already at a lower temperature of ∼250 °C. This is in line with CFM, where the fluorescence from the green laser dominates initially and is then overtaken by the fluorescence from the red and yellow laser. After this initial period, the intensity of fluorescence from all four lasers increases simultaneously during the period of highest C–C bond cleavage rate. This increase can also be seen in the UV-vis spectra (Fig. 3A, bottom).

This indicates that the species observed on ECAT still actively participate in C–C cleavage, while coking occurs simultaneously. In contrast on FCC-cat, the coking rate is the highest only after C–C cleavage has peaked.

**Fig. 4:**
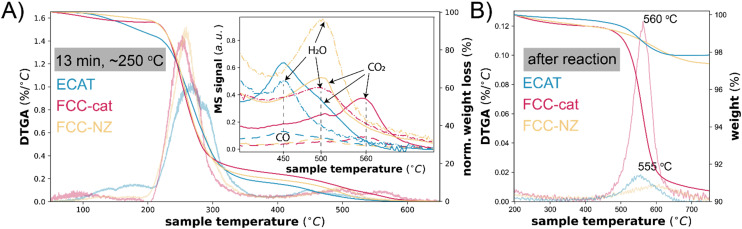
Panel A: Thermogravimetric analysis (TGA) and the derivative of the TGA (DTGA) plot normalized by total weight loss of ECAT, FCC-cat and FCC-NZ samples recovered from the reaction mixture after 13 min. The inset shows the mass spectrometry (MS) trace of the product gasses formed upon burn-off. Panel B shows the TGA and the derivative of the TGA (DTGA) plot normalized by initial weight of ECAT, FCC-cat and FCC-NZ samples recovered after the completed polypropylene (PP) catalytic pyrolysis reaction.

The progression from aromatic intermediates that are likely still participating in the reaction to deactivating polyaromatic species is further studied with thermogravimetric analysis (TGA), burning off remaining species under air. These samples are recovered from the semi-batch reactor after quenching the reaction at relevant time points as described previously.^18^ Partially cleaved polymer, aromatics and coke species can be separated and quantified based on their burn-off temperature, visible when taking the derivative of the normalized weight (DTGA). We assign the burn-off peak between 50–200 °C to aromatic products as their burn-off temperature matches that of naphthalene on these catalysts (Fig. S10†). The burn-off temperature of these species is 50 °C higher for FCC-cat and FCC-NZ than for ECAT The peak between 200–350 °C is assigned to partially cleaved polymer and other aliphatic species and intact polymer (compare this with Fig. S10†). Poly-aromatics and coke deposits are burned off between 400–600 °C. After 13 min of reaction, which corresponds to a reaction temperature of ∼250 °C, and represents the start of C–C cleavage, mainly intact and partially cleaved polymer remains on the catalyst (Fig. 4A and S8†). Over the course of the reaction, the intact and partially cleaved polymers disappear and the burn-off temperature of the aromatic species shifts to higher values, indicating that the species are transformed to coke. The comparison of ECAT to FCC-cat and FCC-NZ reveals naphthalene-like aromatics only on ECAT. In addition, some species with a burn-off temperature between 450 and 560 °C are observed. This could mean that FCC-cat is already more deactivated, in line with the UV-vis data. The coke observed at lower temperature is more hydrogenated than at 560 °C as inferred from the MS trace of the burn-off product gasses (insert Fig. 4A), which show a higher H_2_O/CO_2_ ratio at 500 °C than at 560 °C. Less conjugated species have a higher H/C ratio and can likely still participate in the reaction. The lower the hydrogen content, the more graphitic the structures are and the less likely to participate in the reaction. This interpretation is supported by the fact that after the reaction, only the peak at 560 °C is visible for FCC-cat (Fig. 4B) and it is therefore associated with inactive coke deposits in the zeolite domains.

**Fig. 5:**
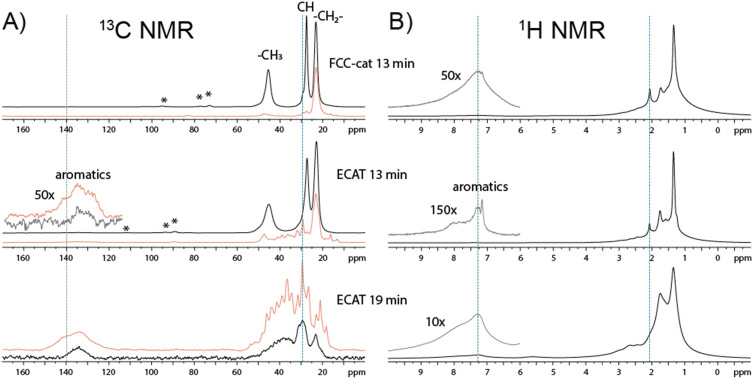
Panel A shows the ^13^C nuclear magnetic resonance (NMR) spectra of the FCC-cat material, recovered after 13 min, and the ECAT material recovered from the polypropylene (PP) reaction mixture after 13 and 19 min of reaction (red – direct excitation, black – cross-polarization). Panel B shows the ^1^H NMR spectra of the FCC-cat material, recovered after 13 min, and the ECAT material recovered from the PP reaction mixture after 13 and 19 min of reaction. Blue and green dotted lines on the spectra in panels A and B correspond to the cross-sections in Fig. 6. Asterisks indicate spinning side band.

In the next stage of our study, we have performed ssNMR experiments, which were found to be consistent with the presence of the substrate PP at the early stages of the reaction, *i.e.*, after 13 min. The ssNMR spectra in Fig. 5A show broad peaks at ∼22, 27, and 45 ppm, related to CH_3_, CH and CH_2_ carbons of polypropylene, respectively (Fig. 5A, top panel),^44^ which then almost fully disappear at the later stage (Fig. 5A, lower panel). At the same time, the ratio of aromatics compared to alkenes and alkanes is increasing, observable both in the ^1^H and ^13^C spectra (Fig. 5). Comparison of ^13^C spectra measured using cross-polarization (*i.e.*, *via* transfer of close-by protons, ^1^H) and direct excitation experiments reveals additional peaks in direct excitation. This indicates the presence of a higher number and amount of deprotonated species at increased conversion of PP (Fig. 5A) with more deprotonated species observed for ECAT after 13 min compared to FCC-cat. An additional shoulder in the aromatics peaks at around 140 ppm could indicate the presence of both protonated and deprotonated aromatics corresponding to the shoulder of burn-off peak in TGA of ECAT and the split into two peaks for FCC-cat (Fig. 4 and S8†). It is conceivable that the more deprotonated aromatic peak corresponds to species that display a higher burn-off temperature (560 °C) in TGA. These species are more conjugated polymeric aromatic species like anthracene with a lower H/C ratio that are likely immobile and deactivate the catalyst.^45^

Notably, ^13^C ssNMR spectra of the FCC-cat do not show noticeable aromatic signals both in cross polarization and direct excitation experiments (Fig. 5A), while the ^1^H ssNMR spectrum contains peaks at aromatic chemical shifts (Fig. 5B). The reason for this discrepancy may be related to the complex nature of our samples and will require further investigation. We also conducted two-dimensional (2-D) ^1^H–^13^C heteronuclear correlation (HETCOR) experiments at ultra-high magnetic field (*i.e.*, 1.2 GHz) using frequency-switched Lee–Goldburg decoupling (FSLG) in the proton (^1^H) dimension.^46^

**Fig. 6:**
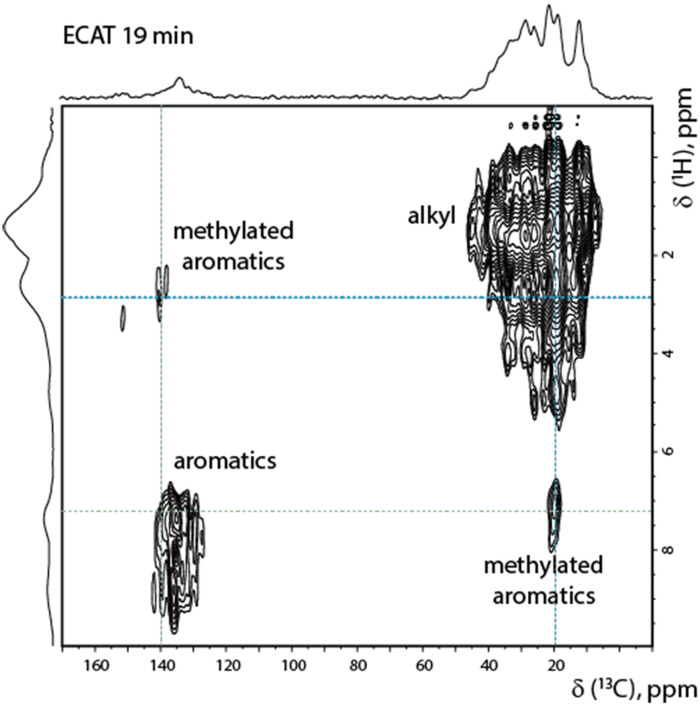
2-D ^1^H–^13^C frequency-switched Lee–Goldburg (FSLG) heteronuclear correlation (HETCOR) spectrum of an *ex situ* ECAT sample recovered from the reaction mixture after 19 min.

The 2-D ^1^H–^13^C FSLG HETCOR NMR spectra show correlations that connect aliphatic protons (horizontal lines, detected in 1-D, Fig. 5B) with aromatic carbons (vertical lines, detected in 1-D, Fig. 5A). This confirms the presence of methylated benzenes and naphthalenes (Fig. 6).

Interestingly, such ssNMR studies were precluded at low-field ssNMR settings due to an insufficient signal-to-noise ratio. We also note that the data shown in Fig. 6, including the multitude of complex resonances both in ^13^C and ^1^H dimensions, confirm the complexity of the molecular species produced at the latter stages of the PP catalytic pyrolysis reaction. A further analysis of the resulting species may be possible using a combination of 3-D spectroscopy and high-sensitivity (dynamic nuclear polarization, DNP) ssNMR setups.

## Conclusions

The combined results of *in situ* IR and UV-vis spectroscopy, *in situ* fluorescence microscopy, and *ex situ* NMR provide relevant information on the various reaction intermediates formed during the catalytic pyrolysis process of polypropylene (PP) over a zeolite-based FCC-catalyst (FCC-cat), the same FCC catalyst without zeolite (FCC-NZ) and metal-containing ECAT. The results obtained suggest that the intermediates consist of methylated aromatic species and are slightly different for the three catalyst materials both in terms of relative amount and type.

The comparison of FCC-cat and FCC-NZ, revealed two different kinds of conjugated poly-aromatic species at early stages of the reaction, which could be assigned to species arising in the matrix and the zeolite domains of the catalyst materials. After the reaction, only species associated with the zeolite domains remain. The presence of the zeolite material enhances the formation of charged alkylated naphthalene species in the early phases of the reaction over the FCC-cat material, which then transform into charged and neutral polyaromatics and deactivating coke species.

It was found that coking is higher on FCC-cat and progresses faster than for the FCC-NZ and ECAT catalyst materials. Interestingly, coking on ECAT proceeds at the same time as C–C cleavage, while on FCC-cat it mainly occurs after C–C cleavage is completed. This could be due to intermediates trapped in the micropores of the zeolite domains, not able to evolve as products and instead transforming into coke deposits, suggesting a two-step process. This suggests that a next generation catalyst material for this reaction would profit from a higher accessibility and a milder acidity compared to the FCC-cat material and shows the great potential of using the discarded ECAT catalyst system for this reaction, and this finding is in line with an earlier study from our group,^42^ in which we have shown the importance of mass transport limitations in FCC-based catalyst materials for polyolefin pyrolysis.

## Data availability

All data is available on Yoda *via* doi https://doi.org/10.24416/UU01-90SYAI.

## Author contributions

The manuscript was written through contributions of all authors. All authors have given approval to the final version of the manuscript. Ina Vollmer and Bert Weckhuysen conceived the ideas for the manuscript. Ina Vollmer designed the *in situ* spectroscopic and microscopic experiments and performed the experiments with the help of Michael Jenks. Sebastian Rejman performed additional thermogravimetric analysis. Marc Baldus and Andrei Gurinov performed nuclear magnetic resonance experiments and wrote the discussion of these results. Ina Vollmer wrote the manuscript with the help of Michael Jenks and Bert Weckhuysen. Bert Weckhuysen, Florian Meirer and Michael Jenks revised and edited the manuscript.

## Conflicts of interest

The authors declare no conflict of interest.

## Supplementary Material

CY-014-D3CY01473H-s001
