# Correction: Unravelling potential reaction intermediates during catalytic pyrolysis of polypropylene with microscopy and spectroscopy

**DOI:** 10.1039/d4cy90029d

**Published:** 2024-04-10

**Authors:** Ina Vollmer, Michael J. F. Jenks, Sebastian Rejman, Florian Meirer, Andrei Gurinov, Marc Baldus, Bert M. Weckhuysen

**Affiliations:** a Inorganic Chemistry and Catalysis Group, Debye Institute for Nanomaterials Science and Institute for Sustainable and Circular Chemistry, Department of Chemistry, Utrecht University Universiteitsweg 99 3584 CH Utrecht The Netherlands b.m.weckhuysen@uu.nl; b NMR Spectroscopy, Bijvoet Center for Biomolecular Research, Department of Chemistry, Utrecht University Padualaan 8 3584 CH Utrecht The Netherlands

## Abstract

Correction for ‘Unravelling potential reaction intermediates during catalytic pyrolysis of polypropylene with microscopy and spectroscopy’ by Ina Vollmer *et al.*, *Catal. Sci. Technol.*, 2024, **14**, 894–902, https://doi.org/10.1039/d3cy01473h.

The published article includes an incorrect version of Fig. 2. The correct version of Fig. 2 is included below.

**Fig. 2:**
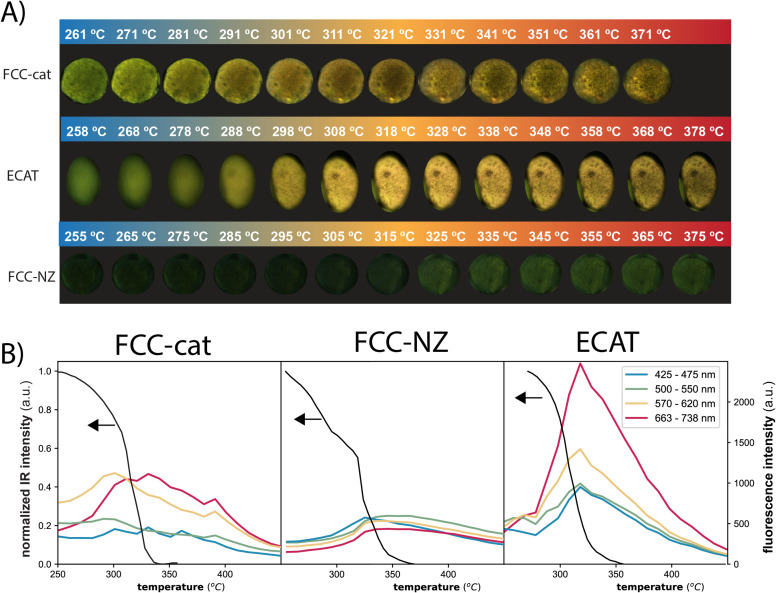
Panel A depicts the *in situ* fluorescence microscopy images of selected FCC-cat (top), ECAT (middle) and FCC-NZ (bottom) particles during the polypropylene (PP) catalytic pyrolysis reaction. All fluorescence microscopy images of all catalyst particles imaged can be found in Fig. S5.† Panel B: The integrated peak area of the C–H bending vibrations measured by *in situ* IR spectroscopy (Fig. 1C) indicates PP breakdown over FCC-cat (left), FCC-NZ (middle) and ECAT (right). The fluorescence intensity in the different wavelength regions is obtained by averaging over all pixels of a sectioned catalyst particle. Evolution of fluorescence for more ECAT particles can be found in Fig. S6.†

The Royal Society of Chemistry apologises for these errors and any consequent inconvenience to authors and readers.

## Supplementary Material

CY-014-D4CY90029D-s001

