# Supplementary material for: Unravelling potential reaction intermediates during catalytic pyrolysis of polypropylene with microscopy and spectroscopy
Source: Catal Sci Technol. 2024 Jan 11;14(4):894–902. doi: 10.1039/d3cy01473h (PMC10876043; doi:10.1039/d3cy01473h)
Supplement: CY-014-D3CY01473H-s001 [file CY-014-D3CY01473H-s001.pdf]

Electronic Supplementary Material (ESI) for Catalysis Science & Technology.
This journal is © The Royal Society of Chemistry 2024

# Unravelling Potential Reaction Intermediates during Catalytic Pyrolysis of Polypropylene with Microscopy and Spectroscopy

Ina Vollmer[1], Michael J.F. Jenks[1], Sebastian Rejman[1], Florian Meirer[1], Andrei Gurinov[2], Marc Baldus[2], Bert M. Weckhuysen[1,*]

[1]Inorganic Chemistry and Catalysis group, Debye Institute for Nanomaterials Science and Institute for Sustainable and Circular Chemistry, Department of Chemistry, Utrecht University, Universiteitsweg 99, 3584 CG Utrecht, The Netherlands

[2]NMR Spectroscopy, Bijvoet Center for Biomolecular Research, Department of Chemistry, Utrecht University, Padualaan 8, 3584 CH Utrecht, The Netherlands

***Materials and Chemicals.*** Isotactic polypropylene (PP) pellets with a 2-8 mm diameter, average $M_w$ ~ 23,000 g/mol and average $M_n$ ~ 7,000 g/mol was obtained from Sigma Aldrich (428116) and carefully ground into smaller pieces for *in-situ* spectroscopy and microscopy measurements using a mortar and crucible. A fresh FCC catalyst (further labeled as FCC-cat, BET surface area: 326.5 $m^2$/g), a FCC-cat without zeolite (further labeled as FCC-NZ, BET surface area: 83.6 $m^2$/g) and an equilibrium catalyst (further labeled as ECAT, BET surface area: 183.2 $m^2$/g) was obtained from Albemarle. The ECAT material was calcined prior to use, heating to 500 °C at 5 °C/min with a hold time of 2 h at 120 °C and 5 h at 500 °C.

**Methods.** For all *in-situ* spectroscopy and microscopy experiments, a 15 ml/min flow of $N_2$ was used and the temperature was increased at 10 °C/min to a final temperature of 530 °C. For isothermal experiments, the temperature was ramped to 250 °C at 100 °C/min. A Linkam FTIR600 stage equipped with $CaF_2$ windows, as shown in **Figure S1**, was used for *in-situ* Fourier transform-infrared (FT-IR) spectroscopy and UV-Visible (UV-Vis) spectroscopy experiments, while a TS1500 Linkam stage, with a very similar internal geometry, was used for the *in-situ* confocal fluorescence microscopy (CFM) experiments.

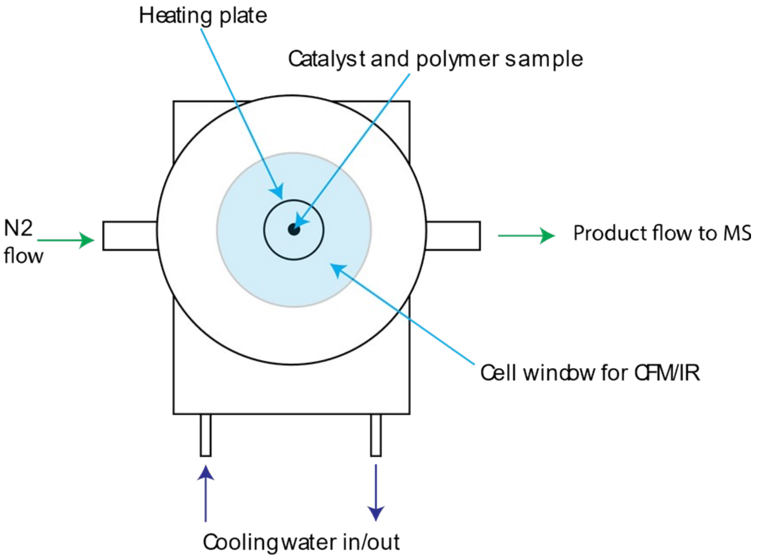


*Figure S1. Schematic of the Linkam reactor used for the in-situ spectroscopy and microscopy experiments during polypropylene (PP) catalytic pyrolysis.*

***In-situ* Fourier transform infrared spectroscopy** (FT-IR) was performed on a PerkinElmer Spotlight 400 infrared microscope equipped with a MCT detector. Spectra were collected in transmission mode with an aperture opening of 30x30 μm using 8 accumulations and a resolution of 2 $cm^{-1}$. A few catalyst particles and PP particles were placed on a round, polished $CaF_2$ window with a 13 mm diameter and 0.5 mm thickness. The background spectrum was taken on a spot without catalyst or plastic. A spectrum was collected every 25s. A polynomial baseline (see python script in supplementary material 2) was subtracted for the C-H stretching and the C-H bending region separately. Other baselines were considered, but did not lead to significant differences in calculated peak areas.

***In-situ* confocal fluorescence microscopy** (CFM) was carried out on a Nikon Instruments A1 in DU4 mode using 403.7, 488, 561.1 and 641.5 nm lasers in combination with a 405/488/561/640 nm 1$^{st}$ dichroic mirror. Images were taken using a 50x objective with a pixel dwell time of 21.6 ns/pixel, a raster size of 1024 pixels, a pinhole size of 66.41 μm and the numerical aperture being set to 0.8. The laser power (LP) and detector sensitivity (HV) settings were kept the same for all images and chosen to utilize the full detector intensity range. The recorded fluorescence intensities were split across four bands of 425-475, 500-550, 570-620 and 663-738 nm and measured by a multi-anode photomultiplier. To obtain the average fluorescence of single particles over time and to exclude the background, the pixels were segmented into particle and background leading to a mask of the particle's cross section using ImageJ. The segmented images were then analyzed using a self-written script in MATLAB (supplementary material 2).

***In-situ* UV-Vis diffuse reflectance spectroscopy** (DRS) measurements were performed with a CRAIC 20/30 PV UV-Vis-NIR micro-spectrophotometer using a 36× objective. A 75 W Xenon lamp was used for illumination. The background spectrum was taken on the catalyst at room temperature prior to the start of the experiment.

***Ex-situ* samples** were produced as described in our previous publication.[1] 2.5 g of PP was loaded together with 1.25 g of catalyst yielding a PP:catalyst ratio of 2:1 into a 50 ml Parr autoclave reactor equipped with a simple stirrer. The stirring speed was 100 rpm. A 50 ml/min flow of $N_2$ was sent through the reactor. The reactor was heated up to 420 °C using a 20 °C/min heating ramp. Reactions were performed at near ambient pressure of about 1.1 atm. The samples were collected after 13 min corresponding to a temperature of about 200 °C quenching the reactor quickly using an ice bath.

***Ex-situ* UV-Vis diffuse reflectance spectroscopy** (DRS) was performed on *ex-situ* samples using a Perkin-Elmer Lambda 950 spectrophotometer equipped with an integrating sphere ("Labsphere") in the 200–800 nm range. The absorption intensity is expressed by the Schuster-Kubelka-Munk equation:

$$F(R_\infty) = (1 - R_\infty)^2/2R_\infty$$

**Solid-state nuclear magnetic resonance** (NMR) measurements the *ex-situ* samples were performed on a 1.2 GHz standard-bore NMR spectrometer (Bruker Biospin). During the experiments, linear drift compensation was active. $^{1}H$ NMR spectra were recorded on a Bruker 1.3 mm triple channel (H/X/Y) CP-MAS probe at an magic angle spinning (MAS) rate of 60 kHz. The $^{13}C$ NMR spectra were obtained using a Bruker 3.2 mm triple resonance ($^{1}H/^{13}C/^{15}N$) CP-MAS probe at MAS frequencies of 18-20 kHz. For $^{13}C$ cross polarisation (CP) spectra, contact times were optimized to 1.5 ms resulting in the maximum signal-to-noise ratio (SNR). For direct detection ($^{1}H$ and $^{13}C$) and $^{13}C$ CP experiments, recycle delays of 6 and 4 s, respectively, were used. Radio frequency (r.f.) power levels used for $^{1}H$ decoupling and $^{1}H/^{13}C$ were set to 75 kHz and 75 kHz/50 kHz, respectively. In the 2-D $^{1}H$-$^{13}C$ FSLG HETCOR experiment,[2] the sample was spun at 18 kHz and a CP contact pulse of 1.5 ms was used. 32 $t_1$ points with 2k transients were recorded in the indirect dimension using Lee-Goldburg decoupling at 75 kHz r.f. field strength. Subsequently, the data were processed using Lorentzian line broadening of 250 Hz. All experiments were recorded at room temperature. Going to lower temperatures (i.e., 260 K) did not alter the results. The $^{13}C$ and $^{1}H$ chemical shifts were referenced to trimethylsilane (TMS) using adamantane as external standard.

**Thermogravimetric analysis** (TGA) measurements of the *ex-situ* samples recovered 13 and 19 min into the reaction as well as spent samples were analyzed using a PerkinElmer TGA 8000 coupled to a Hiden HPR-20 mass spectromer (MS) to determine the total amount of partially cracked plastic and coke as well as their respective burn-off temperature and H/C ratio. About 5 mg of sample was heated to 800 °C (at a rate of 10 °C/min) under a 100 ml/min flow of artificial air (20 vol.% $O_2$ in Ar). The total mass loss was determined by taking the mass at 150 °C as the initial weight, assuming that any prior weight-loss is due to desorption of adsorbed water.

Additional experimental data.

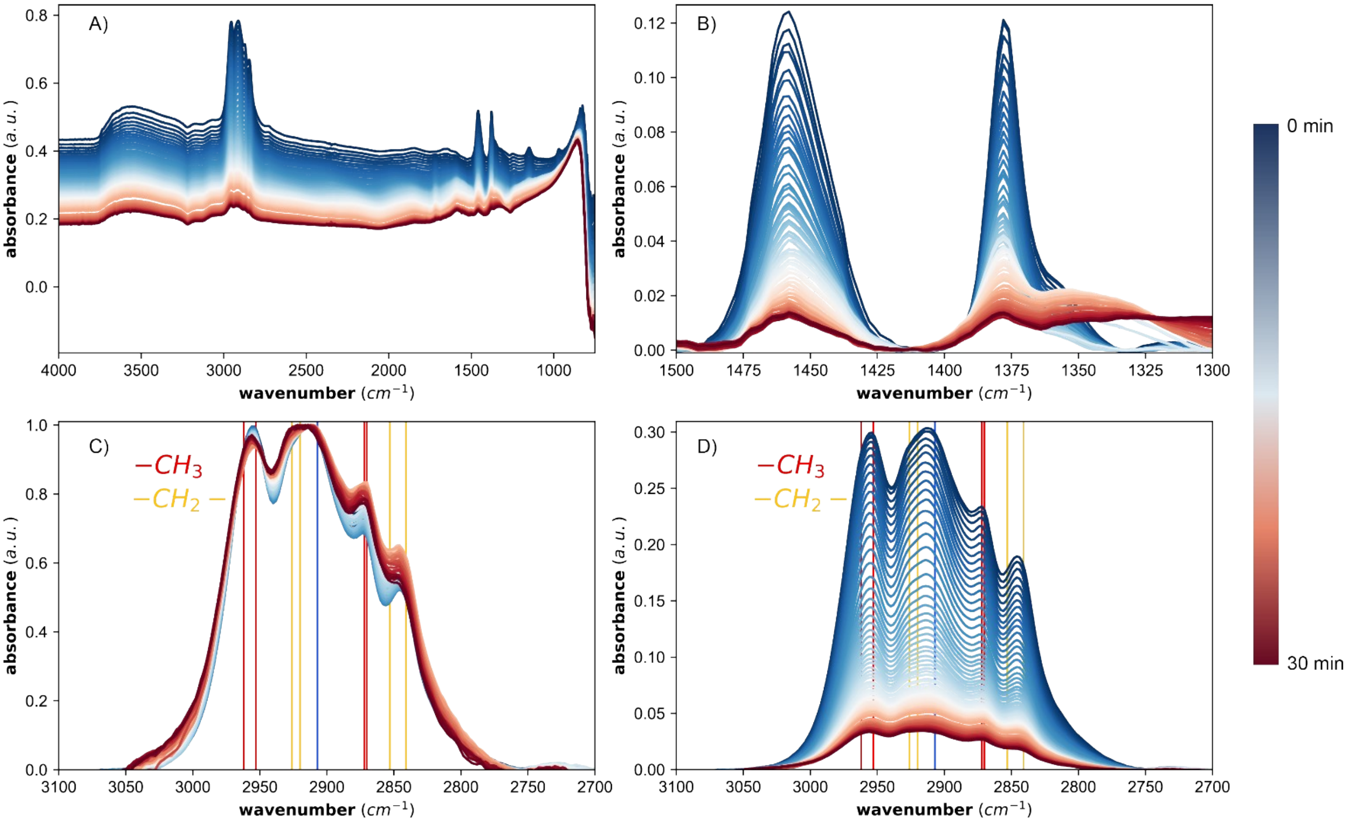


*Figure S2. Infrared (IR) spectra measured during reaction of polypropylene (PP) with a fluid catalytic cracking catalyst (FCC-cat) at 300 °C. Panel A: Raw spectra. Panel B: Background corrected spectra of the C-H bending region. Panel C: Normalized and background corrected spectra of the C-H stretching region. Panel D: Background corrected spectra of the C-H stretching region.*

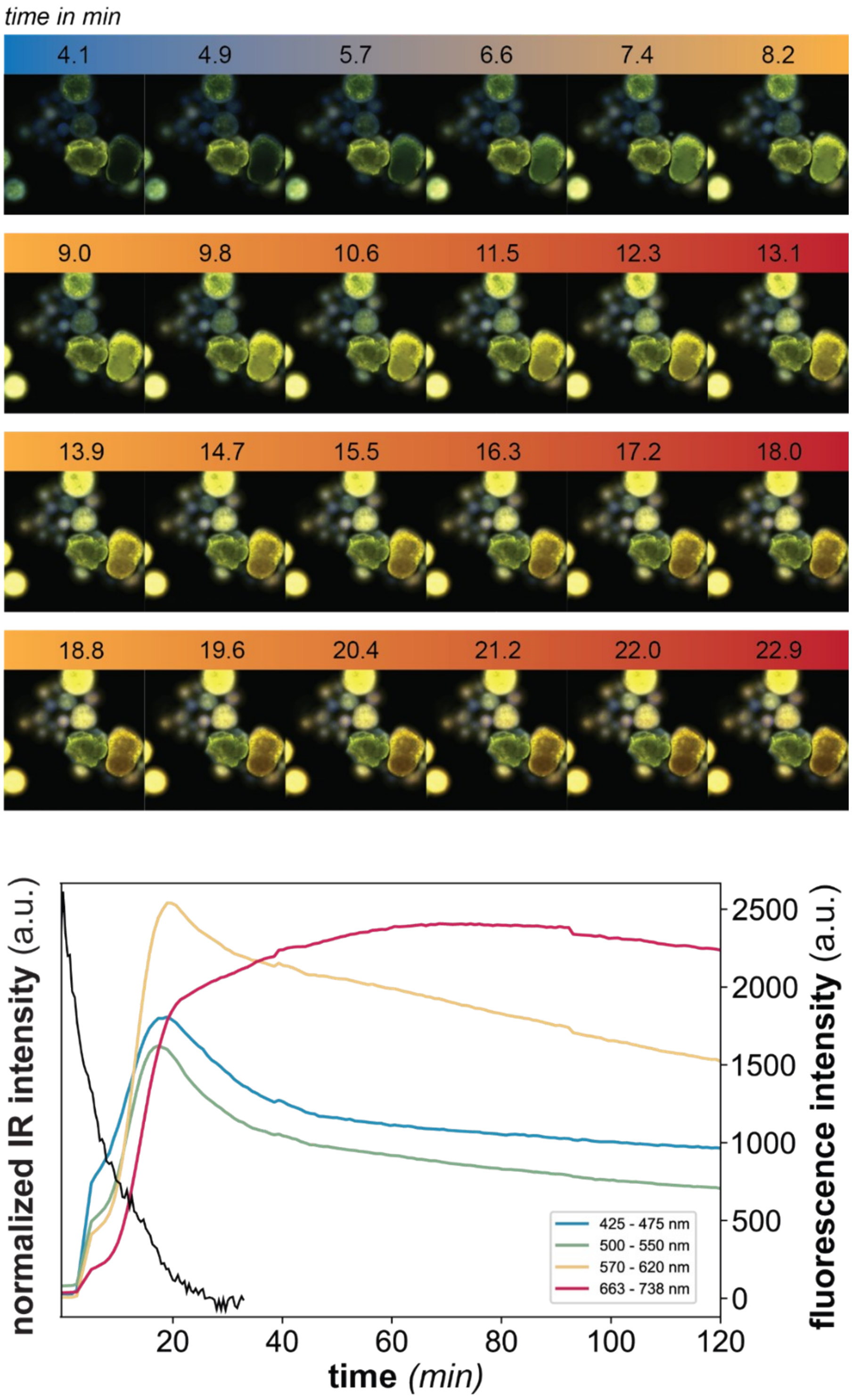


*Figure S3. Top: Confocal fluorescence microscopy (CFM) scans during polypropylene (PP) conversion over a fluid catalytic cracking catalyst (FCC-cat) at 250 °C. Bottom: The integrated peak area of the C-H bending vibrations measured by infrared (IR) spectroscopy (Figure S2) indicates PP breakdown. The fluorescence intensity in the different wavelength regions is obtained by averaging over all pixels of a sectioned catalyst particle.*

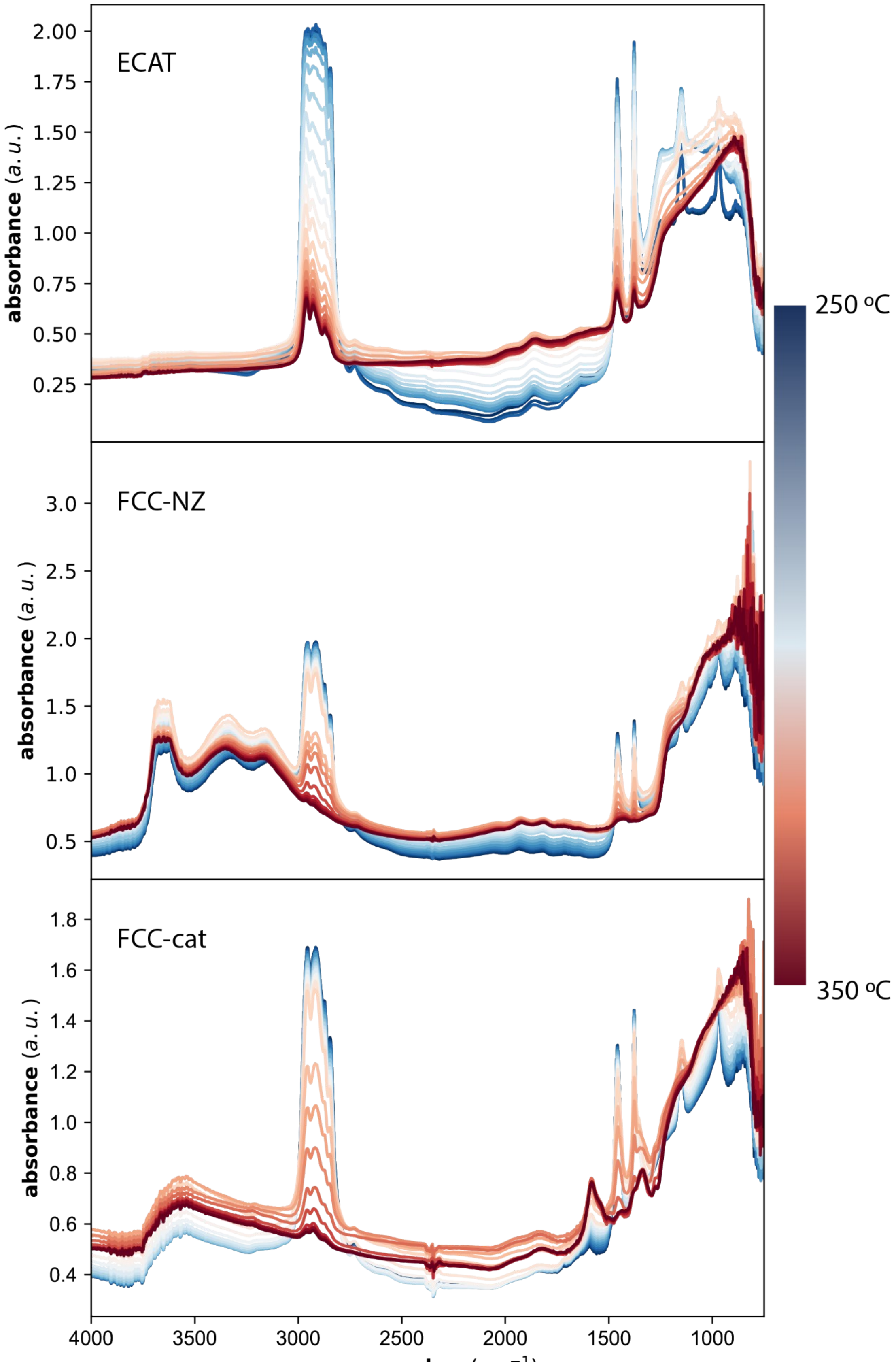

*Figure S4. Raw infrared (IR) spectra measured during reaction of polypropylene (PP) with a fluid catalytic cracking catalyst (FCC-cat), a FCC-cat without zeolite (FCC-NZ) and equilibrium catalyst (ECAT), while ramping the temperature at a rate of 10 ℃/min. The increase in baseline could be due to a general darkening of the sample due to coking.*

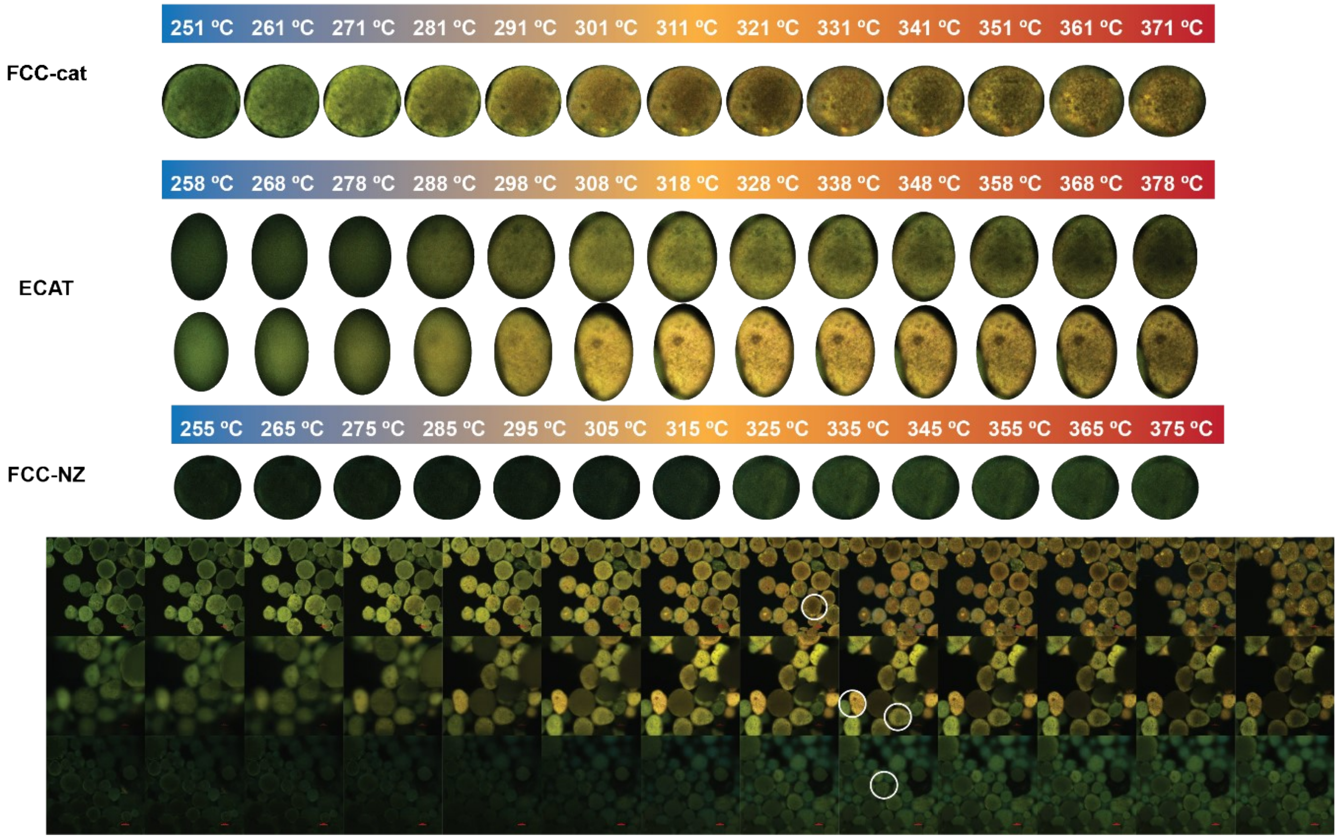


*Figure S5. Confocal fluorescence microscopy (CFM) images of several particles of the family of the fluid catalytic cracking catalyst (FCC-cat), the equilibrium catalyst (ECAT), and the FCC-cat without zeolite (FCC-NZ), taken in the period of highest catalyst activity. The selected catalyst particles are shown at the top with indications of the temperature at which they were taken. The selected catalyst particles are highlighted with white circles in the microscopy images below.*

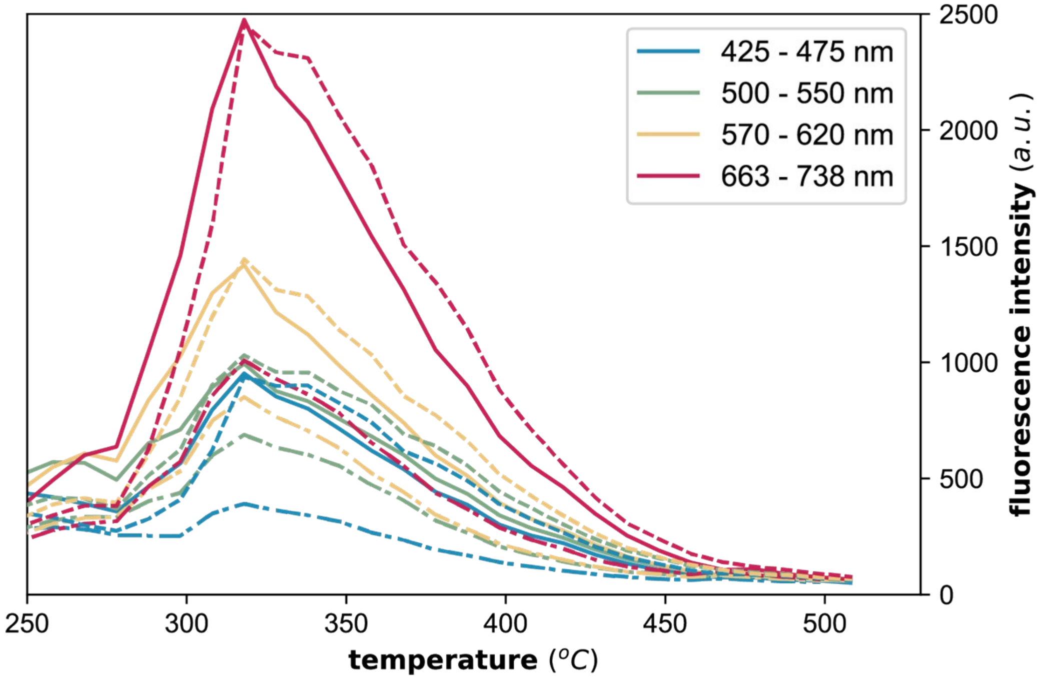


*Figure S6. Different ECAT particles sectioned from the whole confocal fluorescence microscopy (CFM) scan. Dotted-dashed, solid and dashed lines represent particles 1, 2 and 3, as indicated in Figure S4, respectively.*

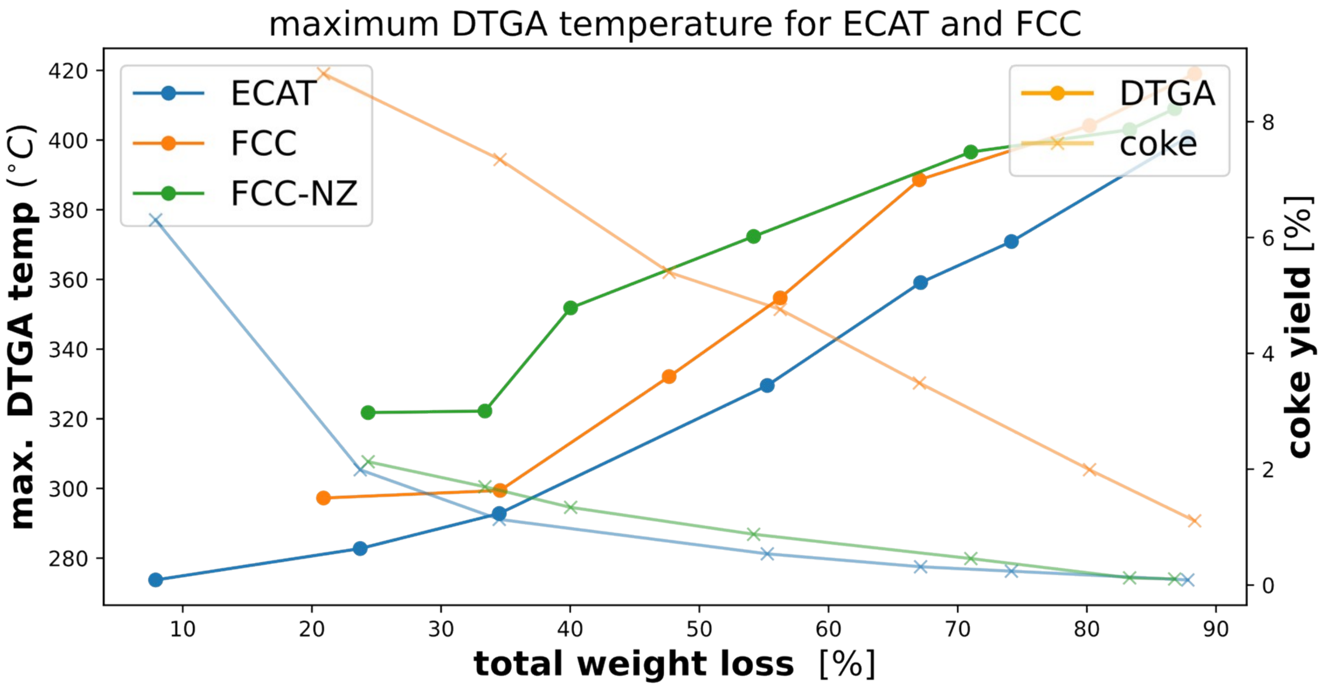


*Figure S7. Results from Thermogravimetric Analysis (TGA) of ECAT, FCC-cat and FCC-NZ with polypropylene under $N_2$ flow and a heating ramp of 10 °C/min. Various polymer to catalyst ratios were used in the experiment. The dots represent the temperatures at which the rate of weight loss, i.e., the differential of the weight loss curves is at its maximum over various polymer to catalyst ratios. The total weight loss is an indication of the percentage of polymer present. The reaction proceeds at the lowest temperature for ECAT and at the highest for FCC-NZ over a wide range of polymer to catalyst ratios. Coke yields are indicated by crosses, corresponding to the right axis. Most coke remains on FCC-cat, while similar coke yields are observed on FCC-NZ and ECAT.*

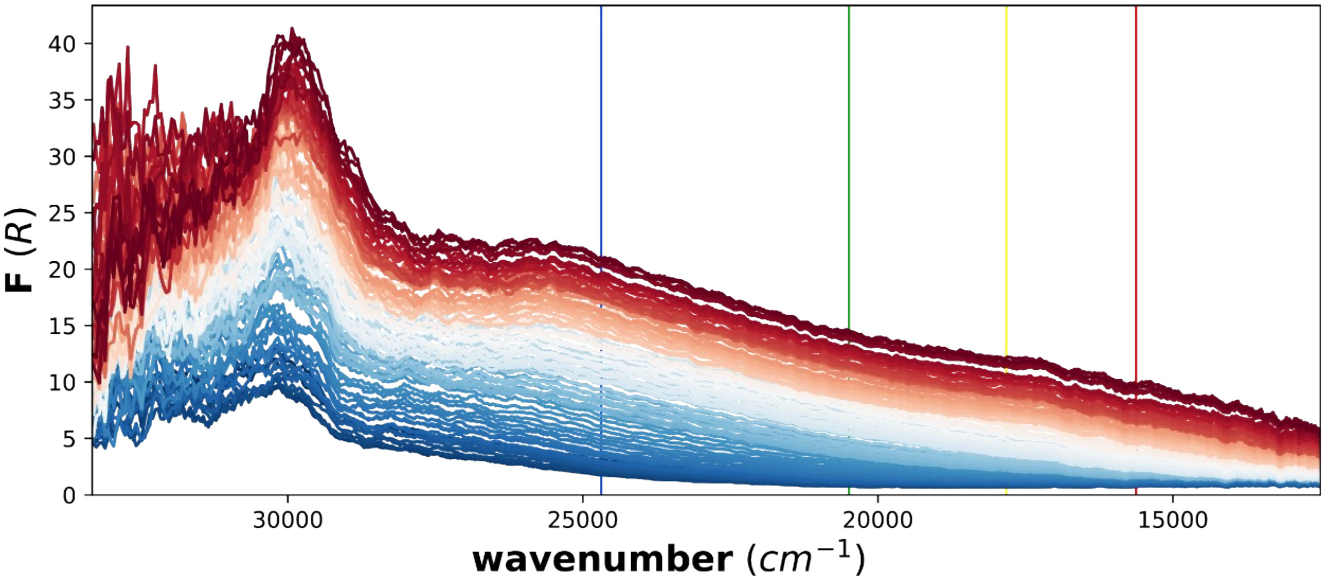


*Figure S8. In-situ UV-Vis spectroscopy of FCC-cat heated under 15 ml/min flow of $N_2$ with the same temperature program as the other in-situ experiments but without the addition of polypropylene (PP) as a blank experiment.*

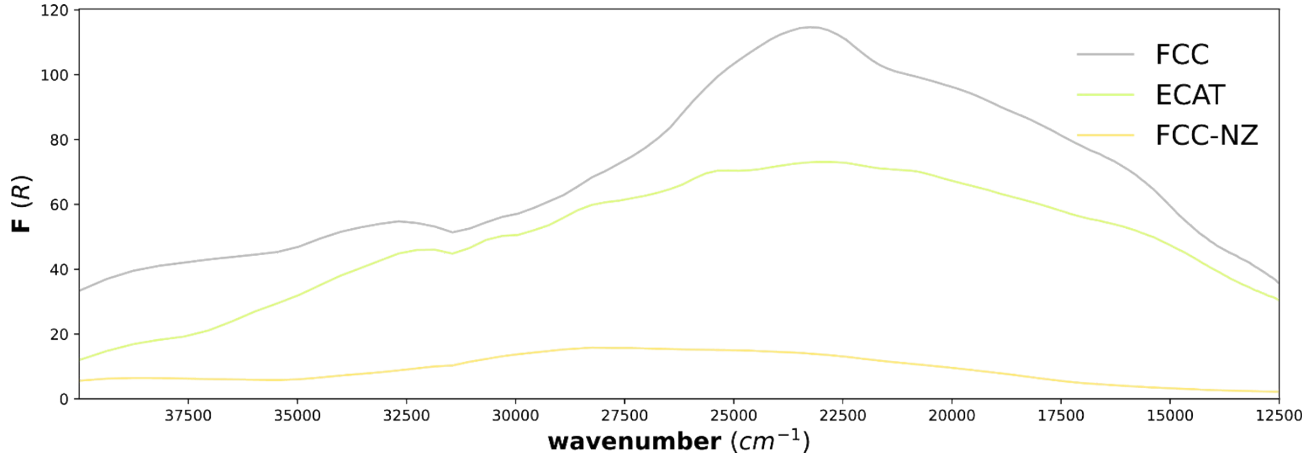


*Figure S9. UV-Vis diffuse reflectance spectroscopoy (DRS) measurements on ex-situ catalyst samples, recovered after 13 min of the polypropylene (PP) pyrolysis reaction.*

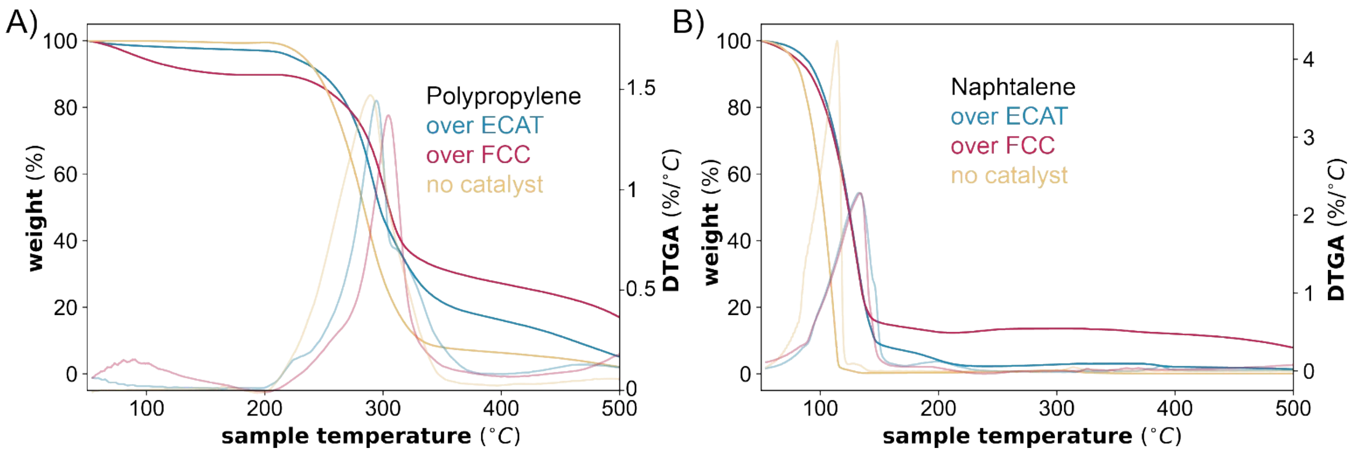


*Figure S10. Thermogravimetric Analysis (TGA) normalized to overall weight loss and differential TGA (DTGA) curves recorded during burn off of Panel A: polypropylene and Panel B: napthalene over ECAT, FCC-cat and without catalyst.*

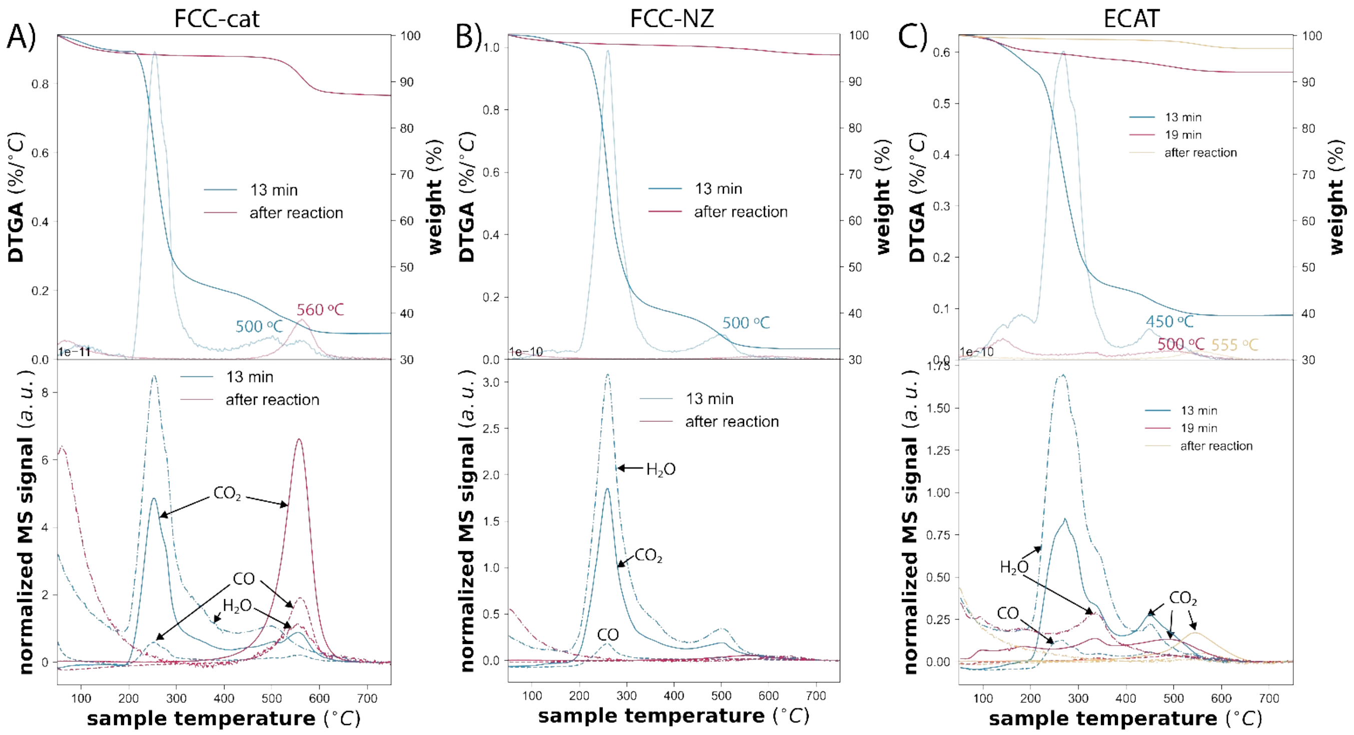


*Figure S11. Thermogravimetric Analysis (TGA) normalized to starting weight measured on catalyst materials recovered quenching the polypropylene (PP) pyrolysis reaction at relevant time points and after reaction together with the mass spectrometry (MS) traces recorded simultaneously (bottom). Panel A: the FCC-cat material recovered after 13 min and after reaction. Panel B: the FCC-NZ material recovered after 13 min and after reaction. Panel C the ECAT material recovered after 13 min, 19 min and after reaction.*

## References


1. Vollmer, I., Jenks, M. J. F., González, R. M., Meirer, F. & Weckhuysen, B. M. Plastic Waste Conversion over a Refinery Waste Catalyst. *Angew. Chem. Int. Ed.* **60**, 16101–16108 (2021).

2. van Rossum, B.-J., Förster, H. & de Groot, H. J. M. High-Field and High-Speed CP-MAS $^{13}C$ NMR Heteronuclear Dipolar-Correlation Spectroscopy of Solids with Frequency-Switched Lee–Goldburg Homonuclear Decoupling. *J. Magn. Reson.* **124**, 516–519 (1997).
